# AI-driven epidemic intelligence: the future of outbreak detection and response

**DOI:** 10.3389/frai.2025.1645467

**Published:** 2025-07-30

**Authors:** Jasleen Kaur, Zahid Ahmad Butt

**Affiliations:** ^1^School of Public Health Sciences, Faculty of Health, University of Waterloo, Waterloo, ON, Canada; ^2^National Research Council Canada, Digital Technologies Research Centre, Ottawa, ON, Canada

**Keywords:** epidemic intelligence, artificial intelligence, outbreak detection, large language models, pandemic preparedness, real-time surveillance

## Abstract

Epidemic intelligence, the process of detecting, verifying, and analyzing public health threats to enable timely responses, traditionally relies heavily on manual reporting and structured data, often causing delays and coverage gaps. The growing frequency of emerging infectious diseases highlights the urgency for more rapid and accurate surveillance methods. This perspective proposes a forward-looking conceptual framework for AI-driven epidemic intelligence, emphasizing the transformative potential of integrating large language models (LLMs), natural language processing (NLP), and optimization-based resource allocation strategies. While existing AI-driven systems have shown significant capabilities during the COVID-19 pandemic, several challenges remain, including real-time adaptability, multilingual data handling, misinformation, and public health policy alignment. To address these gaps, we propose an integrated, real-time adaptable LLM-based epidemic intelligence system, capable of correlating cross-source data, optimizing healthcare resource allocation, and supporting informed outbreak response. This approach aims to significantly improve early warning capabilities, enhancing forecasting accuracy, and strengthen pandemic preparedness.

## Introduction

Epidemic intelligence is the process of detecting, verifying, and analyzing public health threats to enable timely responses (ASSET, 2015). The growing frequency of emerging infectious diseases highlights the need for rapid and accurate surveillance (MacIntyre et al., 2022). Traditional epidemic surveillance often relies on manual analysis of structured data, primarily from official public health reports. However, such conventional methods frequently experience significant delays and coverage gaps, especially in regions with limited healthcare infrastructure. These limitations highlight the urgent need for an automated, AI-driven system that can enable real-time analysis of diverse data and multilingual data streams (Friesema et al., 2015; Abat et al., 2016; Choi et al., 2016; Murray and Cohen, 2017; Jung et al., 2019). Additionally, incorporating data from multiple internet-based sources, such as social media, online news, and search queries, has shown promise in improving epidemic forecasting accuracy (Li et al., 2022). This article is presented as a perspective, aiming to propose a forward-looking conceptual framework for integrated AI-driven epidemic intelligence. The goal is to synthesize insights from large language models (LLMs), outbreak forecasting, and emergency department optimization to improve surveillance and response.

## AI-driven epidemic intelligence

AI-driven epidemic intelligence has emerged as a transformative tool for public health surveillance, offering the ability to analyze structured and unstructured data sources in real time. The Large Language Models (LLMs) (Consoli et al., 2024; Deiner et al., 2024), advanced AI systems capable of understanding and generating human language at scale, and Natural Language Processing (NLP) (Al-Garadi et al., 2022), a subfield of AI focused on enabling machines to interpret human language, help extract meaningful insights from multilingual data streams, overcoming barriers that previously limited comprehensive global surveillance. These technologies can rapidly process open-source information, including news reports, social media trends, and web searches, significantly reducing detection time compared to manual surveillance methods (Brownstein et al., 2023; MacIntyre et al., 2023; Giri and Gupta, 2024).

During the COVID-19 pandemic, the adoption of AI-driven epidemic intelligence grew significantly, highlighting how machine learning can enhance traditional surveillance by identifying early warning signs for further analysis (MacIntyre et al., 2023). Furthermore, AI has played an important role not only in outbreak detection but also in vaccine development and pandemic preparedness, highlighting its potential for global health security (From outbreak to vaccine: Artificial intelligence’s contribution to pandemic preparedness, 2025).

While these systems have improved outbreak detection, they remain fragmented and reactive, often struggling with misinformation filtering, lack of cross-source integration, and real-time adaptability. Addressing these limitations is crucial for AI to effectively support public health infrastructure. Many existing AI systems are designed for either detection or response, but not both, and they struggle to dynamically update as an outbreak evolves. Additionally, public health agencies have been slow to adopt AI-driven tools due to trust issues, integration difficulties, and policy constraints. Addressing these limitations is critical for AI to effectively support public health infrastructure.

An increasingly important challenge is the risk of unintended cross-source influence. While many current AI models are designed to analyze each data stream, such as clinical reports, social media, or news articles, independently, there are situations where unintended cross-source analysis can occur. For example, signals from non-healthcare data sources or social platforms may influence outbreak assessments, particularly when engagement algorithms amplify health related content. Such data may not reflect true epidemiological changes but rather social perceptions, demographic shifts, or online behavior patterns. Although these non-traditional sources can provide useful early signals, their inclusion without proper validation introduces the risk of misleading conclusions if they are interpreted out of context.

To mitigate these risks, AI systems aligned with public health goals should include features such as credibility scoring systems, source validation checks, and anomaly detection algorithms that flag content potentially shaped by virality or engagement-driven amplification. This is especially important for signals from platforms like social media, where high-engagement posts may not represent actual health threats. At the same time, excluding all non-healthcare data can create blind spots, especially in regions where formal reporting is limited. Limiting AI surveillance only to secure, structured healthcare data sources such as those standardized under HL7 FHIR (Health Level Seven Fast Healthcare Interoperability Resources) (Mandel et al., 2016; Overview - FHIR v5.0.0, 2025) may reduce early warning capabilities. A balanced, hybrid approach that combines structured healthcare data with carefully filtered open-source inputs can provide richer, more contextual insights while minimizing the risk of misinformation.

Another key limitation of existing AI-driven surveillance systems is that they process each data source independently, leading to a failure in capturing critical cross-source correlations. This disconnect prevents current models from forming a comprehensive, contextual understanding of potential outbreaks (McClymont et al., 2024). For example, a local hospital may report a surge in respiratory cases, while social media discussions in the region highlight concerns about an unknown flu-like illness, and at the same time, the World Health Organization may request information from authorities regarding unusual pneumonia cases. Traditional surveillance systems might flag each of these independently but fail to connect them into a coherent warning. An LLM-based epidemic intelligence system, could synthesize these signals, generating a contextual insight such as: “*A potential outbreak is escalating, with multiple independent sources confirming rising cases of an unknown respiratory illness*.” This ability to correlate seemingly disparate sources can significantly enhance early warning systems, making them more effective in detecting and contextualizing emerging threats (Consoli et al., 2024; Deiner et al., 2024).

## AI-driven disease surveillance: applications and challenges

Several AI-driven disease surveillance platforms have been developed to detect emerging outbreaks in real time. HealthMap (Brownstein et al., 2008; HealthMap | Flu map | contagious disease surveillance | virus awareness, 2025), an automated system launched in 2006, monitors global online news for infectious disease reports. EPIWATCH (MacIntyre et al., 2022; EPIWATCH - Home, 2025; EPIWATCH – rapid epidemic intelligence | UNSW Research, 2025), an AI-driven early warning system, scans public health reports and social media, providing alerts ahead of official announcements. Similarly, Epitweetr (epitweetr tool, 2020; MacIntyre et al., 2023), developed by the European Centre for Disease Prevention and Control (ECDC), continuously monitors Twitter for signs of infectious disease events. Other platforms include ProMED-mail (Madoff, 2004; Woodall and Calisher, 2025; Home - ProMED, 2025), a moderated global disease reporting network, and BlueDot (BlueDot: The world’s most trusted infectious disease intelligence, 2025; Tracking the Coronavirus Pandemic with AI: BlueDot featured on 60 Minutes, 2025), a commercial analytics company that detected the initial COVID-19 outbreak before public health agencies raised alarms. These platforms analyze vast amounts of data, including news feeds, social media discussions, and official health reports, using machine learning and NLP techniques.

Early digital surveillance efforts such as Google Flu Trends (Butler, 2013) highlighted both the potential and pitfalls of AI-driven epidemic monitoring. While it initially showed promise by using search query patterns to estimate influenza prevalence, it later significantly overestimated flu levels due to model overfitting and failure to account for media-driven behavioral changes. This example, as discussed by Lazer et al. (2014) emphasizes the importance of rigorous validation, model adaptability, and contextual awareness in the development of modern AI-driven epidemic intelligence systems. During the COVID-19 pandemic, AI-driven surveillance platforms proved their value and effectiveness. For example, BlueDot (BlueDot: The world’s most trusted infectious disease intelligence, 2025) identified an unusual pneumonia outbreak in Wuhan (Bogoch et al., 2020) before official confirmation. Similarly, Canada’s Global Public Health Intelligence Network (GPHIN) (Mykhalovskiy and Weir, 2006; Carter et al., 2018) historically scanned online sources in multiple languages to detect global outbreaks.

Recent studies (Consoli et al., 2024; Du et al., 2024, 2025; Giri and Gupta, 2024; Deiner et al., 2025; Rama et al., 2025; AI Model Offers New Approach to Infectious Disease Forecasting, 2025) have significantly expanded the capabilities of AI-driven epidemic intelligence, particularly in multilingual NLP, hybrid modeling, and LLM-enhanced forecasting. PandemicLLM (Du et al., 2025), a multi-modal LLM architecture for outbreak forecasting, outperforms traditional time-series models by integrating policy, genomic, and behavioral data. Another recent system (Consoli et al., 2024) demonstrated the use of multilingual LLM ensembles for extracting outbreak-related signals from unstructured health reports and news sources, improving both detection speed and contextual accuracy. SIR-INN (Rama et al., 2025), a physics-informed neural network, integrates real-time flu data into a modified SIR framework, offering both interpretability and predictive accuracy. Additionally, EpiLLM (Gong et al., 2025; Jiao et al., 2025), a dual-branch architecture, fuses spatio-temporal epidemic trends with mobility data to generate localized disease spread predictions. These recent contributions reinforce the importance of integrated, AI-driven approaches and further validate the conceptual foundation proposed in this paper.

However, despite these technological advances, significant challenges remain. Many platforms generate large volumes of unfiltered alerts, requiring human analysts to validate and interpret findings. This verification process can slow response times and reduce the real-time advantage of AI. Additionally, surveillance systems must filter misinformation and distinguish credible signals from misleading social media content, a task that remains complex due to the variability of language and evolving terminology. Another challenge is the adaptability of AI models. Outbreak indicators can shift rapidly, and AI systems must continuously update their models to capture emerging trends. Handling multilingual and multi-source data remains an ongoing hurdle, as NLP models require refinement to improve accuracy across different languages and cultural contexts. Furthermore, many public health agencies hesitate to fully integrate AI-driven surveillance tools into their decision-making processes due to concerns about reliability, interoperability with existing workflows, and data privacy regulations. Addressing these challenges requires the development of more robust NLP techniques, improved data validation methods, and stronger collaboration between AI developers and public health professionals.

## Epidemiological modeling and AI-enhanced outbreak forecasting

While early detection is critical, outbreak prediction and transmission modeling are essential for effective response planning. Epidemiological modeling plays an important role in outbreak intelligence by projecting disease spread and guiding intervention strategies (Murray and Lopez, 1996; Keeling and Eames, 2005). Traditional epidemiological models, example SIR (Susceptible-Infectious-Recovered) and SEIR (Susceptible-Exposed-Infectious-Recovered), simulate transmission dynamics using differential equations. However, these models rely on fixed assumptions and historical parameters, limiting adaptability during evolving outbreaks (Holmdahl and Buckee, 2020). AI-driven epidemiological models integrate machine learning techniques such as recurrent neural networks and graph neural networks, analyzing complex temporal and spatial patterns. Hybrid AI models incorporating real-time data sources such as mobility trends, web searches, and social media significantly improve forecasting accuracy and adaptability (Ginsberg et al., 2009; Santillana et al., 2015; Yang et al., 2015; Holmdahl and Buckee, 2020; McGough et al., 2020; Liu et al., 2024).

However, a gap remains, epidemiological models often function in isolation, failing to integrate outbreak predictions with healthcare resource allocation. Accurate forecasts alone do not translate into effective responses unless embedded within real-time decision-support systems.

## AI-based resource allocation and decision-support systems

While AI has improved outbreak detection, existing epidemic surveillance systems do not integrate real-time emergency department (ED) resource optimization (Sahu, 2023; Gawande et al., 2025; Siddiqui et al., 2025; Rise of the Machines - Artificial Intelligence in Healthcare Epidemiology | Current Infectious Disease Reports, 2025). Traditionally, hospital resource allocation, such as ICU beds, medical supplies, and vaccines; has been managed separately from surveillance, which leads to delays in response and inefficient distribution of critical resources. AI-driven epidemic intelligence must evolve beyond early detection to include real-time healthcare resource optimization in EDs, where rapid decision-making is critical.

By analyzing ED wait times and patient inflow trends, AI can help predict congestion, adjust triage strategies, and improve resource distribution. For example, during an outbreak, AI could track increases in respiratory cases in EDs, allowing hospitals to proactively allocate additional personnel, beds, or medical supplies. AI-based decision support could also simulate different intervention strategies, such as adjusting triage thresholds, reassigning medical staff, or optimizing emergency response protocols to prevent overcrowding and delays in care. Embedding scenario-based decision support within epidemic intelligence systems can significantly enhance response effectiveness. AI-driven models could provide real-time recommendations for ED management based on emerging outbreak patterns, allowing hospitals to take proactive rather than reactive measures.

However, real-world adoption remains limited due to regulatory constraints, privacy concerns, interoperability challenges with hospital systems, and policy misalignment. Addressing these barriers requires stronger collaboration between AI developers, healthcare providers, and public health agencies to ensure seamless integration into clinical workflows. By linking epidemic intelligence with ED decision support, healthcare systems can improve patient outcomes, reduce wait times, and enhance emergency response capacity during outbreaks, ultimately strengthening hospital resilience and public health preparedness.

## Public health policies and AI integration

Public health policies play a crucial role in shaping the adoption of AI-driven epidemic intelligence. However, regulatory constraints, interoperability challenges, and data privacy concerns limit full integration into public health systems. The Canada Communicable Disease Report highlights that while AI and novel data sources can enhance public health surveillance, challenges such as limited real-world implementation, interoperability barriers, and privacy concerns restrict their effectiveness in synthesizing outbreak-related insights across multiple sources (Canada PHA, 2024a). Additionally, GDPR (EU) and HIPAA (US) impose strict data protection regulations that limit real-time health data exchange, affecting AI’s ability to detect emerging threats efficiently (Panch et al., 2025; Riel, 2025). While these regulations are critical for privacy protection, they also highlight the need for policies that balance data security with real-time epidemic response (Brownstein et al., 2023b).

Interoperability remains a key challenge, as healthcare databases, surveillance platforms, and AI models often function in isolation, preventing effective cross-source data synthesis. Canada’s Global Public Health Intelligence Network (GPHIN) (Mykhalovskiy and Weir, 2006; Carter et al., 2018; Canada PHA, 2024a) has played a critical role in early outbreak detection by scanning global online sources for potential health threats. However, challenges in integrating GPHIN with broader public health decision-making processes have highlighted the need for improved interoperability, real-time adaptability, and AI explainability (Government of Canada, 2018). Canada’s Public Health Data Strategy (Canada PHA, 2022, 2024b) emphasizes modernizing public health infrastructure, ensuring accountable governance, and adopting standardized data frameworks, but full integration remains limited due to fragmented data systems and slow policy adaptation.

Successful integration can be facilitated by adopting interoperable data standards, such as HL7 FHIR (Mandel et al., 2016; Overview - FHIR v5.0.0, 2025), and establishing application programming interfaces (APIs) that effectively bridge new AI models with legacy public health surveillance databases. However, real-world FHIR adoption remains uneven, with challenges including inconsistent EHR implementations, limited technical infrastructure, and lack of alignment between clinical and public health reporting systems. Overcoming these barriers will require coordinated efforts across government agencies, healthcare vendors, and policymakers. Strengthening data-sharing policies, improving AI explainability, and ensuring ethical AI governance are critical to enhancing AI-driven epidemic intelligence (Morley et al., 2020; Panch et al., 2025; Does “AI” stand for augmenting inequality in the era of covid-19 healthcare? | The BMJ, 2025). Policymakers’ hesitations, often stemming from the perceived opacity of AI’s “black box” models, can be addressed by enhancing AI explainability through techniques such as hybrid models that combine interpretable algorithms with deep learning, and utilizing explainability frameworks like SHAP (SHapley Additive exPlanations). Engaging policymakers directly through workshops and trainings customed to enhance their understanding of AI-driven insights can further build trust and acceptance.

Given the known risks of LLM hallucination, bias, and privacy vulnerabilities, it is critical that AI-driven epidemic intelligence systems incorporate safeguards such as human-in-the-loop validation, explainability techniques, and source credibility scoring. Additionally, federated learning and privacy-preserving data architectures can support secure deployment in line with health data regulations such as GDPR and HIPAA. These considerations are essential to building trust, ensuring reliability, and aligning with ethical public health practices.

Canadian efforts align with global initiatives such as the CDC’s Center for Forecasting and Outbreak Analytics (CDC, 2025) and the WHO’s Pandemic Intelligence Hub (who_hub.pdf, 2025), reinforcing the importance of AI-driven epidemic intelligence systems that integrate real-time data analytics, interoperability, and public health decision support. Addressing these policy and integration challenges is essential to enabling a more effective, transparent, and adaptable epidemic intelligence infrastructure.

## Research gap and proposed solution

While AI-driven epidemic intelligence has made significant advancements, critical gaps remain in integrating AI-driven epidemic intelligence into real-time decision-making frameworks. Currently, epidemic intelligence systems often operate independently, addressing either detection, prediction, or resource allocation separately rather than offering a fully integrated approach. Many AI-driven surveillance tools lack real-time adaptability, struggling to update dynamically as outbreaks evolve, which compromises the timeliness and accuracy of public health interventions. Additionally, existing AI models typically fail to correlate insights across multiple data sources, missing crucial contextual patterns that could provide more comprehensive outbreak assessments. This limitation, frequently described as the “connecting dots” problem, restricts public health systems’ capacity to fully recognize emerging threats and limits their ability to proactively respond to converging evidence from multiple streams of information.

Moreover, public health agencies remain slow to adopt AI-driven epidemic intelligence tools due to concerns regarding their reliability, interoperability with existing healthcare workflows, and data privacy issues. Ethical and governance considerations, including algorithmic bias, transparency, fairness, and accountability, further complicate the effective implementation and acceptance of AI-driven solutions in public health contexts (Morley et al., 2020).

To bridge these critical gaps, this perspective article proposes an integrated Large Language Model (LLM)-based epidemic intelligence system, unifying outbreak detection, AI-driven predictive modeling, and optimization-based emergency department (ED) resource allocation within a cohesive framework. The novelty of this proposed approach lies in its real-time adaptability, ensuring continuous model updates as outbreaks evolve, thereby addressing dynamic disease surveillance challenges. Furthermore, integrating cross-source data fusion capabilities through advanced NLP techniques enables the AI system to synthesize information across diverse data streams, resolving the “connecting dots” issue by capturing critical contextual cross-source relationships and delivering actionable, contextualized insights.

This integrated system could help public health authorities move beyond passive detection to proactive outbreak response, ultimately improving pandemic preparedness and resilience. By addressing real-time adaptability, cross-source data integration, and decision support, this research solution aims to advance the state of the art in AI-driven epidemic intelligence and contribute to more effective, equitable, and responsive public health strategies.

The proposed solution also emphasizes linking epidemic forecasts directly with ED-specific resource allocation. Unlike conventional public health surveillance systems, the proposed integrated system could proactively inform public health decisions by continuously optimizing ED triage strategies, wait time reduction, and emergency bed availability. Scenario-based decision support embedded within epidemic intelligence frameworks would evaluate intervention strategies in real-time, allowing ED administrators to dynamically adjust patient triage, staffing, and bed allocation based on emerging outbreak patterns.

Additionally, leveraging advanced multilingual NLP capabilities within LLMs significantly enhances global surveillance capacity, enabling accurate, culturally sensitive interpretation of diverse language sources. This multilingual approach substantially improves outbreak detection accuracy and comprehensiveness, particularly benefiting low-resource regions that often lack robust structured-data infrastructures.

Finally, addressing ethical concerns and governance issues, this proposed research prioritizes transparency, fairness, and explainability in AI methodologies. Ensuring human oversight, independent validation and audits, open communication of AI performance metrics, and clear stakeholder explanations will foster public trust, regulatory alignment, and broader adoption of AI-driven recommendations among public health stakeholders.

**Figure 1:**
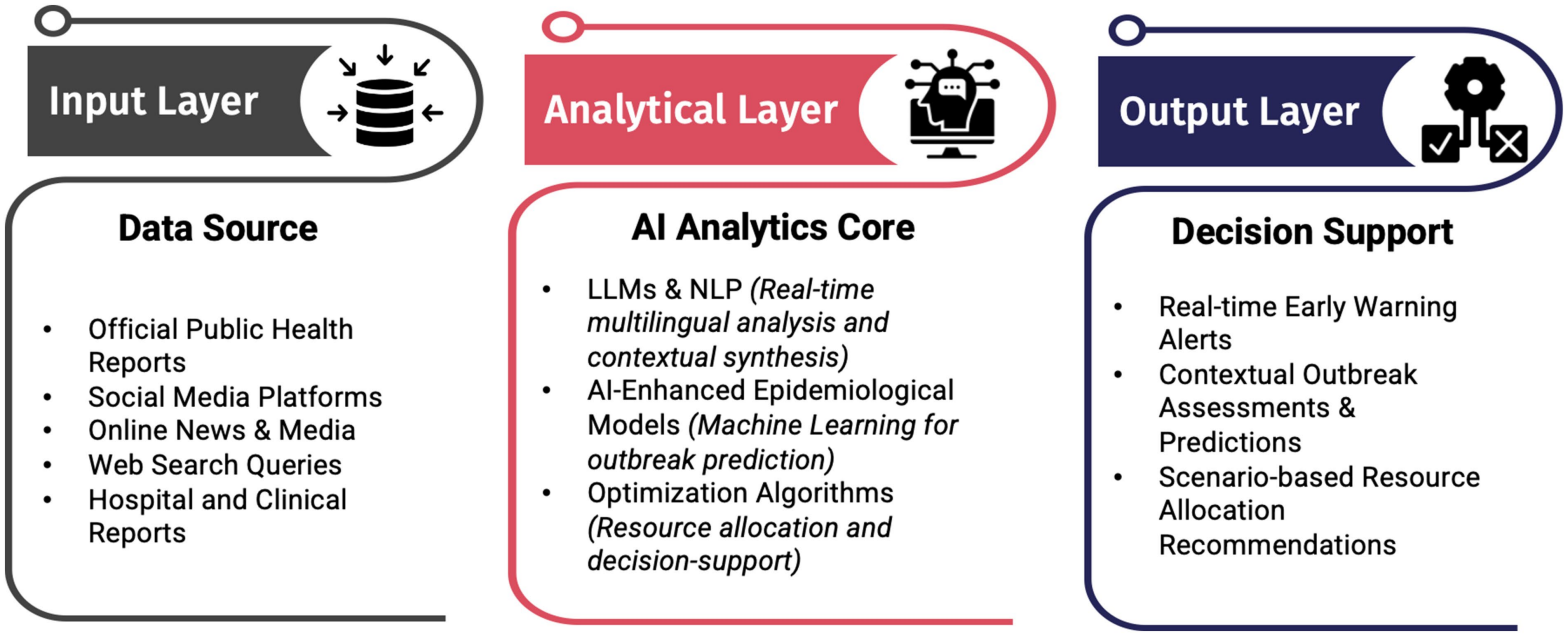
Conceptual architecture of the proposed integrated AI-driven epidemic intelligence framework, highlighting the flow from multisource data input through AI-driven analytics to real-time decision support for public health and emergency departments.

This proposed integrated system could help public health authorities move beyond passive detection to proactive outbreak response, improving pandemic preparedness and resilience. The proposed system, shown in Figure 1, highlights the interconnectedness of multiple data sources (input layer), advanced analytical components such as large language models, epidemiological modeling, and optimization algorithms (analytical layer), and real-time decision-support outputs (output layer), all integrated to enable real-time adaptability, cross-source data fusion, enhanced emergency department capacity planning, and response efficiency.

## Conclusion

AI-driven epidemic intelligence represents a paradigm shift in public health surveillance and response. Integrating advanced AI technologies, including large language models for multilingual surveillance, predictive analytics for outbreak forecasting, and optimization algorithms for healthcare resource management, into a single, cohesive decision-support system can significantly enhance early detection, forecasting accuracy, and outbreak response effectiveness. Overcoming existing barriers such as multilingual data handling, misinformation management, real-time adaptability, and policy integration through the proposed solution will facilitate faster, more accurate, and equitable responses to future pandemics, ultimately strengthening global health preparedness and resilience. Putting clear strategies into practice for AI integration, improving AI explainability to reduce policymakers’ hesitation, and actively building public trust through transparency and oversight will directly address current concerns and significantly enhance the practical adoption and acceptance of AI-driven epidemic intelligence systems. Future work should focus on validating the proposed system through real-world simulation, comparative analysis with existing platforms, and empirical testing in hospital-based outbreak scenarios. Additionally, stakeholder engagement, policy harmonization, and interdisciplinary collaboration will be critical for scaling AI-driven epidemic intelligence globally.

## Data Availability

The original contributions presented in the study are included in the article/supplementary material, further inquiries can be directed to the corresponding author.
